# Whole-Genome Sequences of Two Klebsiella pneumoniae Strains (Sequence Types 23 and 35) from Wildlife

**DOI:** 10.1128/mra.00140-22

**Published:** 2022-05-17

**Authors:** A. Cornacchia, A. Chiaverini, G. Centorotola, M. Di Domenico, A. Cocco, M. Ancora, C. Cammà, N. D’Alterio, C. E. Di Francesco, F. Pomilio

**Affiliations:** a Istituto Zooprofilattico Sperimentale dell’Abruzzo e del Molise “G. Caporale”, Teramo, Italy; b Università degli Studi di Teramo, Veterinary Medicine Faculty, Teramo, Italy; University of Maryland School of Medicine

## Abstract

This report describes the draft genomes of two Klebsiella pneumoniae strains that were isolated from two wild boars collected during epidemiological surveillance and monitoring of wild fauna in the Abruzzo and Molise regions. The strains belonged to sequence type 23 (ST23) and ST35, which are frequently reported in clinical cases.

## ANNOUNCEMENT

The broader ecological distribution *of* Klebsiella pneumoniae strains and the increasing contact between humans and wildlife highlight the need for intensive monitoring in the environment (1, 2).

In this study, two strains were isolated (2021.TE.17 and 2021.TE.18, from intestine and brain, respectively). Ten grams of an analytical portion was enriched in 90 mL of buffered peptone water (Biolife Italiana, Monza, Italy) at 37°C ± 1°C for 24 ± 1 h. The enrichment broth was streaked on Simmons citrate agar with inositol (SCAI) (3) and incubated at 44°C ± 1°C for 48 ± 1 h. Typical yellow colonies were picked up, subcultured on nutrient agar (Microbiol & C., Cagliari, Italy), incubated at 37°C ± 1°C for 24 ± 1 h, and then identified by matrix-assisted laser desorption ionization–time of flight mass spectrometry (MALDI-TOF MS) (Bruker Daltonik, Bremen, Germany).

DNA was extracted with the DNeasy blood and tissue kit (Qiagen, Hilden, Germany), according to the method described by Portmann et al. (4), and was quantified using the Qubit double-stranded DNA (dsDNA) high-sensitivity (HS) assay kit (Thermo Fisher Scientific, Waltham, MA). The purity of DNA was then checked with a Nanodrop ND-1000 spectrophotometer (Thermo Fisher Scientific).

Genomic libraries were prepared using a DNA preparation kit (Illumina, San Diego, CA) and sequenced on the NextSeq 500 platform (Illumina) with a setting of 300 cycles (150 paired-end reads). Trimming and quality control were performed on a total of 10,652,142 reads (2021.TE.17) or 948,102 reads (2021.TE.18) using Trimmomatic v.036 (5) and FastQC v.0.11.5 (6), respectively. *De novo* assembly was accomplished by SPAdes v.3.11.1 (7) and quality checked by QUAST v.4.3 (8).

The draft assembly size of strain 2021.TE.17 was 5,544,108 bp, arranged in 114 contigs, with a sequencing depth of 255×, an *N*_50_ value of 273,596 bp, and a G+C content of 57.3%. The assembled draft genome of strain 2021.TE.18 was 5,608,041 bp in length, organized in 95 contigs, with a sequencing depth of 22×, an *N*_50_ value of 286,034 bp, and a G+C content of 57.2%. The genome annotations of 2021.TE.17 and 2021.TE.18, using the NCBI Prokaryotic Genome Annotation Pipeline (PGAP) (9), returned 5,270 coding sequences (CDSs), 8 rRNAs, and 70 tRNAs and 5,417 CDSs, 12 rRNAs, and 66 tRNAs, respectively.

The species was confirmed as K. pneumoniae for both strains by Kleborate (10) hosted on the Pathogenwatch platform (11). The sequence types (STs) were determined using the multilocus sequence typing (MLST) scheme hosted on the BIGSdb platform (12), which identified ST23 (2021.TE.17) and ST35 (2021.TE.18).

β-Lactam (*SHV-33*), tetracycline (*tetA*), sulfonamide (*sul2*), and aminoglycoside (*strA* and *strB*) resistance genes were detected in the 2021.TE.18 strain by querying the Kleborate database. In contrast, only *SHV-11* was identified in the 2021.TE.17 strain. Moreover, Kleborate allowed assignment of virulence scores of 5 (*ybt1*, *clb2*, *iuc1, iro1*, and hypermucoidy *rmp1*) and 4 (*ybt5* and *iuc3*) to 2021.TE.17 and 2021.TE.18, respectively. PlasmidFinder (13) allowed detection of RepKleb in 2021.TE.17 and IncFIB(K) and IncFIB(pKPHS1) plasmid replicons in 2021.TE.18. Default parameters were used for all bioinformatic tools mentioned above.

The multidrug resistance and virulence determinants recognized suggest particular attention to K. pneumoniae strains circulating in wild animals. The draft genomes presented here may contribute to confirm the links between humans, animals, and the environment in a One Health approach.

The diagnostic samples were collected during national epidemiological surveillance and monitoring activities involving wild fauna. Therefore, this work was conducted on carcasses of animals that were dead due to anthropic activities (gunshot wounds or impact with vehicles) and subjected to necropsy. For this reason, institutional ethics committee review was not applicable.

### Data availability.

These whole-genome projects have been deposited in GenBank under accession numbers JAJFVQ000000000 and SRR17027470 for strain 2021.TE.17 and JAJFVP000000000 and SRR17027469 for strain 2021.TE.18. The version described in this paper is the first version. The BioProject accession number is PRJNA774508. The BioSample accession numbers are SAMN22567294 and SAMN22567295.
